# Morbidity, outcomes and cost-benefit analysis of wildlife rehabilitation in Catalonia (Spain)

**DOI:** 10.1371/journal.pone.0181331

**Published:** 2017-07-18

**Authors:** Rafael Angel Molina-López, Santi Mañosa, Alex Torres-Riera, Manel Pomarol, Laila Darwich

**Affiliations:** 1 Centre de Fauna Salvatge de Torreferrussa, Catalan Wildlife Service, Forestal Catalana, Santa Perpètua de Mogoda, Spain; 2 Departament de Biologia evolutiva, ecologia i ciències ambientals—Institut de Recerca de la Biodiversitat (IRBio), Universitat de Barcelona, Facultat de Biologia, Avinguda Diagonal, Barcelona, Spain; 3 Departament de Sanitat i Anatomia Animals, Facultat de Veterinària, Universitat Autònoma de Barcelona (UAB), Cerdanyola del Vallès, Spain; 4 Centre de Recerca en Sanitat Animal (CReSA), Institut de Recerca i Tecnologia Agroalimentàries (IRTA), Campus UAB, Cerdanyola del Vallès, Spain; University of Lleida, SPAIN

## Abstract

**Background:**

There are few studies of careful examination of wildlife casualties in Wildlife Rehabilitation Centers. These studies are essential for detecting menaces to wild species and providing objective criteria about cost-benefit of treatments in those centers. The release rate is considered the main outcome indicator, but other parameters such as length of stay at the center and a cost-benefit index expressed as number of released animals per euro and day, could be used as reliable estimators of the rehabilitation costs.

**Methodology:**

A retrospective study based on 54772 admissions recorded from 1995–2013 in the database of the Wildlife Rehabilitation Center of Torreferrussa (Catalonia, NW Spain) assessed the morbidity, outcomes and cost-benefits of the rehabilitation practices.

**Results:**

Three hundred and two species were included: 232 birds (n = 48633), 37 mammals (n = 3293), 20 reptiles (n = 2705) and 13 amphibians (n = 141). The most frequent causes of admission were: 39.8% confiscation of protected species (89.4% passerines), 31.8% orphaned young animals (35.3% swifts, 21.7% diurnal raptors and owls) and 17.4% trauma casualties (46.7% raptors and owls). The highest proportion of releases was found in the captivity confiscation category [87.4% passerines (median time of stay: 12 days)], followed by the orphaned category [78% owls (66 days), 76.5% diurnal birds of prey (43 days), 75.6% hedgehogs (49 days), 52.7% swifts (19 days) and 52% bats (55 days)]. For the trauma group, 46.8% of releases were hedgehogs (44 days) and 25.6% owls (103 days). As regards the cost-benefit index, the trauma casualties and infectious diseases had the worse values with 1.3 and 1.4 released animals/euro/day respectively, and were particularly low in raptors, waders, marine birds and chiroptera. On the contrary, captivity (4.6) and misplacement (4.1) had the best index, particulary in amphibian, reptiles and passerines.

**Conclusions/significance:**

Cost-benefit studies including the release rate, the time of stay at the center and the cost-benefit index should be implemented for improving management efficiency of the Wildlife Rehabilitation Centers.

## Introduction

Wildlife Rehabilitation is defined as the treatment and temporary care of injured, diseased and displaced indigenous animals, and the subsequent release of healthy animals to appropriate habitats in the wild [1]. Benefits and limitations of this activity have been thoroughly discussed [2,3]. Moreover, there is a consensus on the value of Wildlife Rehabilitation Centers (WRC) in monitoring the health of ecosystems, detecting threats to wild animal populations and improving of the wild animal welfare [4]. There are thousands of these centers worldwide working with a huge diversity of species and with different levels of specialization, different management protocols and diverse budgets. Namely, benchmarking can be a very complicated task among the high heterogeneity of WRC.

The information recorded in the WRC can become a material of great value for conservation, providing evidence of the natural or anthropogenic menaces for the species. Although there are many morbidity studies in wildlife [5,6,7,8], most of them are restricted to specific zoological categories, and studies covering a wide variety of animal species or covering long periods of time are still scarce [9,10,11]. Moreover, reviews of wildlife casualties providing objective criteria about cost-benefit of the casualties’ treatments are also poorly reported in the literature [12,13].

In the present study, we analyze 54772 cases attended at the WRC of Torreferrussa, comprising 302 different wild species in a 19-year-long period, including primary causes of admission (morbidity), release and death rates (as main outcome indicators), the rehabilitation stay period in the center and a cost-benefit index as an approach to calculate the rehabilitation costs. In order to improve our understanding and the efficiency of the rehabilitation process, our objective was to evaluate the relative importance and temporal variation of different primary causes of admission during the study period, as well as to evaluate the effectiveness of the rehabilitation in different taxa and in relation to these primary causes.

## Materials and methods

### Study design

A retrospective study was performed using the original medical records of the animals admitted at the WRC of Torreferrussa (Catalonia, North-East Iberian Peninsula). The center receives animals from Catalonia, mainly from the North and Central areas. Catalonia is a state of Spain located at the Mediterranean subregion of the western Palearctic (3°19’-0°9’ E and 42°51’-40°31 N). Wild animals admitted alive from 1995 to 2013 were included in the analyses. Any domestic or exotic species, non-wild born individuals or captive born cases, as well as any case with a total lack of information about the patient were excluded from the analysis.

The rehabilitation center is under the direction of the Catalan Wildlife-Service, who stipulates the management protocols and Ethical Principles according to the Catalan [14] and Spanish legislation [15].

### Animal classification

For each individual admitted to the center we recorded species, sex and age. For statistical analysis, species were grouped in the following broader taxonomic categories: Amphibians (including Anura and Caudata), Reptiles (including Testudines and Squamata), Mammals (including Carnivora, Artiodactyla, Chiroptera, Rodentia, Lagomorpha and Insectivora), Diurnal birds of prey (Accipitriformes), Owls (Strigiformes), Marine Birds (including Procellariformes, Suliformes, Charadriformes), Herons and allies (including Pelecaniformes, Ciconiformes and Phoenicopteriformes), Waders (Charadrifomes), Other Aquatic birds (including Anseriformes, Gruiformes and Podicipediformes), Swifts (Apodiformes), Passerines (Passeriformes) and Other birds (including Columbiformes, Galliformes, Coraciiformes, Caprimulgiformes, Piciformes, Bucerotiformes, Cuculiformes and Otidiformes). Sex was determined when possible by inspection in dimorphic species or by gonadal examination at necropsy. The age was categorized as “first calendar year” and “>1 year calendar” for all the animal groups [16].

### Morbidity analysis

The categories and subcategories of the causes of admission were based on the primary diagnoses [17,18]. Briefly, causes were grouped in the following main categories: “Trauma” associated with an anthropogenic activity or structure (collision–with vehicles, buildings or other human structures, power lines, fences-, electrocution, gunshot, and unknown trauma), “Orphaned” (chicks, fledging or young animals, supposedly abandoned by their parents or fallen from their nest), “Captivity” (animals maintained in captivity for more than 6 months and/or confiscated by the rangers or the police due to poaching or illegal pet trade), “Infectious disease” (infectious or parasitic disease, based in clinical diagnoses or by confirmation of a pathogenic microorganism), “Metabolic or nutritional disease” (low body condition, weakness, and other diseases grouped by organ system), “Misplacement” (animals accidentally found in wrong places, such as buildings or other human made structures, water bodies or vehicles); “Other causes” [(including Natural injuries or casualties (predation, entangled by plants…), intoxication (confirmation of toxic agents), and a miscellaneous of causes as oiled birds, bycatch, predation…)] and “Undetermined”(when it was not possible to assign the cause to any of the above mentioned categories)]. Primary causes were also grouped in two categories, according to the human contribution, as follows: anthropogenic (gunshot, captivity, intoxication, electrocution, collisions with power lines, vehicles, human structures and fences, oiled, unknown trauma, misplacement, and other) and natural (metabolic or nutritional, infectious disease and orphaned).

A prognostic scoring was defined according to the severity of the illness or injury at the moment of the admission, including the following categories: 1, apparently healthy; 2, mild weakness or thinning, uncomplicated fractures; 3, severe (including dehydration, open fractures, deep wounds) and 4, very severe (major injuries, emaciation, paralysis, blindness, respiratory distress).

### Outcome analysis

After admission a bird could follow four different outcomes: 1) Euthanasia, which was humanely assisted death applied to animals with low prognosis or low quality of life, 2) Unassisted death, which occurred during treatment of some animals, 3) Release to the wild, of successfully healed individuals with good perspectives to adapt in the wild, and 4) Captivity, for non-releasable animals that were kept permanently captive, due to their poor prognosis of survivability in the wild. According to these categories, four outcome indicators of the final dispositions of the rehabilitation process were considered and expressed as a rate between the number of cases of each category by the total number of admissions in a given period of time [13]: 1) Release rate (R_r_: number of animals released to the wild/total number of animals admitted), 2) Euthanasia rate (E_r_: number of animals euthanized/total number of animals admitted), 3) Mortality rate (M_r_: number of animals experiencing unassisted death during treatment/total number of animals admitted), and 4) Captivity rate (C_r_: number of animals kept permanently captive/total number of animals admitted).

### Cost- benefits estimator

The time of the rehabilitation stay (T_s_) in the center was used as the basic estimator or approach for assessing the cost of the rehabilitation process. This parameter (T_s_) was defined as the length of time that the animal was retained in the center, that is, the period in days from the date of admittance to the date of release or death of the animal. In order to study the T_s_, the percentiles 10 (P_10_) and 90 (P_90_) of this variable were selected as cut-off points.

On the other hand, a cost-benefit index was calculated as a ratio between the number of released animals and the total cost in euros (cost per day (euros) * number of animals) for each taxonomic group and cause of admission and prognostic category. The daily cost per animal was assumed the same for all species along the rehabilitation process. In order to estimate the daily cost per animal, we selected data from 2008 to 2012. Thus, the average expenses of the WRC (334.583 euros, including staff), were divided by the product [(number of cases/year)* 365 days)], obtaining a value of 0.19 euros/animal/day. Therefore, this estimator expresses the number of released animals per euro of expenses per day of stay, along the period of the study.

### Other variables

The variable “People that brought the animal” included: Rangers, Others Police Authorities, Private individuals, Others and Unknown.

### Statistical analysis

Descriptive statistics, normality test and inferential analyses were done using 95% of confidence intervals (95%CI) with SPSS Advanced Models ™ 15.0 (SPSS Inc. 233 South Wacker Drive, 11th Floor Chicago, IL 60606–6412). Morbidity and outcome studies were analyzed for variation among the different groups of animals, seasons or among years of the study. Comparisons of the median were evaluated using the U-Mann-Whitney and Kruskal-Wallis test. Chi-square or Fisher exact tests were used for comparisons between the causes of admission, outcomes rates, sex, age and taxonomical categories. Linear regression model was used to estimate the trend of the causes of admission and final dispositions during the period of study. Mean, Confidence Intervals of 95% (CI95%), Median (P_50_) and Percentiles 10 and 90 (P_10_; P_90_) were provided for the descriptive analysis of the cost-benefit of the rehabilitation process.

## Results

### Animal data section

The revision process was done in 65335 admission reports. Most of the animals were brought to the WRC by the competent authorities, such as the Rangers (75%), other police authority’s (9%) and by private citizen (12%). The final sample for the study included 54772 cases (10563 cases were excluded for no fulfilling the inclusion criteria described above, including dead admissions for forensic investigation). The final study population included 302 different species (Table 1): most of them (88.5% of cases) considered as protected species by the Catalan legislation and represented about 60% of the total species reported in Catalonia [19,20,21]. Some species are included in the Spanish threatened list [22] as ‘‘in danger of extinction” or “vulnerable” such as *Testudo hermanni*, *Testudo graeca*, *Botaurus stellaris*, *Aythya nyroca*, *Gypaetus barbatus*, *Chlidonias niger*, *Calonectris diomedea*, *Phalacrocorax aristotelis*, *Ardeola ralloides*, *Circus pygargus*, *Aquila fasciata*, *Milvus milvus*, *Neophron percnopterus*, *Pandion haliaetus*, *Tetrao urogallus*, *Tetrax tetrax*, *Larus audoinii*, *Aegolius funereus*, *Phoenicurus phoenicurus*, and *Miniopterus schreibersi*. However, both groups of threatened species represented a small percentage (3%) of the total cases admitted for rehabilitation.

**Table 1:** Species included in the study.

**Birds**								
Anseriformes	n	%	Charadriifromes	n	%	Strigiformes	n	%
*Anas platyrhynchos*	526	92.4	*Larus michahellis*	518	47.4	*Athene noctua*	1655	31.8
*Aythya nyroca* ^*§*^	9	1.6	*Scolopax rusticola*	162	14.8	*Otus scops*	1476	28.3
*Tadorna tadorna*	8	1.4	*Larus ridibundus*	131	12.0	*Strix aluco*	1064	20.4
*Anas querquedula*	4	0.7	*Burhinus oedicnemus*	47	4.3	*Tyto alba*	678	13.0
*Anser anser*	4	0.7	*Larus audouinii* ^*§*^	35	3.2	*Bubo bubo*	209	4.0
*Anas crecca*	3	0.5	*Fratercula arctica*	29	2.7	*Asio otus*	111	2.1
*Anas penelope*	3	0.5	*Vanellus vanellus*	26	2.4	*Asio flammeus*	15	0.3
*Netta rufina*	3	0.5	*Alca torda*	23	2.1	*Aegolius funereus*^*§*^	1	0.0
*Somateria mollissima*	3	0.5	*Himantopus himantopus*	23	2.1	Total	5209,0	100.0
*Anas acuta*	1	0.2	*Charadrius alexandrinus*	17	1.6	Accipitriformes	n	%
*Anas strepera*	1	0.2	*Larus melanocephalus*	12	1.1	*Falco tinnunculus*	1994	40.6
*Aythya ferina*	1	0.2	*Sterna sandvicensis*	10	0.9	*Buteo buteo*	1150	23.4
*Aythya fuligula*	1	0.2	*Sterna hirundo*	9	0.8	*Accipiter nisus*	637	13.0
*Cygnus olor*	1	0.2	*Charadrius dubius*	8	0.7	*Accipiter gentilis*	344	7.0
*Tadorna ferruginea*	1	0.2	*Gallinago gallinago*	4	0.4	*Falco peregrinus*	161	3.3
Total	569	100	*Calidris minuta*	3	0.3	*Gyps fulvus*	96	2.0
Podicipediformes	n	%	*Numenius phaeopus*	3	0.3	*Pernis apivorus*	88	1.8
*Tachybaptus ruficollis*	19	54.3	*Pluvialis apricaria*	3	0.3	*Falco naumanni*	76	1.5
*Podiceps nigricollis*	10	28.6	*Tringa totanus*	3	0.3	*Circaetus gallicus*	67	1.4
*Podiceps cristatus*	6	17.1	*Calidris alpina*	2	0.2	*Falco subbuteo*	61	1.2
Total	35	100.0	*Haematopus ostralegus*	2	0.2	*Circus aeruginosus*	44	0.9
Phoenicopteriformes	n	%	*Larus minutus*	2	0.2	*Hieraaetus pennatus*	42	0.9
*Phoenicopterus roseus*	38	100.0	*Rissa tridactyla*	2	0.2	*Circus pygargus*^*§*^	40	0.8
Gruiformes	n	%	*Tringa ochropus*	2	0.2	*Milvus migrans*	39	0.8
*Gallinula chloropus*	96	58.2	*Actitis hypoleucos*	1	0.1	*Hieraaetus fasciatus*^*§*^	19	0.4
*Rallus aquaticus*	27	16.4	*Calidris alba*	1	0.1	*Circus cyaneus*	15	0.3
*Porphyrio porphyrio*	13	7.9	*Calidris canutus*	1	0.1	*Falco columbarius*	10	0.2
*Fulica atra*	9	5.5	*Calidris ferruginea*	1	0.1	*Milvus milvus*^*§*^	10	0.2
*Crex crex*	8	4.8	*Charadrius hiaticula*	1	0.1	*Pandion haliaetus*	4	0.1
*Porzana porzana*	8	4.8	*Charadrius morinellus*	1	0.1	*Aquila chrysaetos*	3	0.1
*Grus grus*	2	1.2	*Chlidonias hybrida*	1	0.1	*Aegypius monachus*	2	0.0
*Porzana parva*	2	1.2	*Chlidonias niger*^*§*^	1	0.1	*Buteo rufinus*	2	0.0
Total	165	100.0	*Larus fuscus*	1	0.1	*Falco vespertinus*	2	0.0
Pelecaniformes	n	%	*Limosa lapponica*	1	0.1	*Gypaetus barbatus*^*§*^	2	0.0
*Ardea cinerea*	195	41.1	*Limosa limosa*	1	0.1	*Neophron percnopterus*^*§*^	2	0.0
*Bubulcus ibis*	109	23.0	*Philomachus pugnax*	1	0.1	Total	4910	100.0
*Egretta garzetta*	70	14.8	*Stercorarius parasiticus*	1	0.1	Procellariformes	n	%
*Ixobrychus minutus*	55	11.6	*Sterna albifrons*	1	0.1	*Hydrobates pelagicus*	4	44.4
*Ardea purpurea*	17	3.6	*Tringa glareola*	1	0.1	*Calonectis diomedea*^*§*^	3	33.3
*Nycticorax nycticorax*	12	2.5	*Tringa nebularia*	1	0.1	*Puffinus yelkouan*	2	22.2
*Botaurus stellaris*^*§*^	11	2.3	*Uria aalge*	1	0.1	Total	9	100.0
*Platalea leucorodia*	2	0.4	Total	1093	100.0			
*Ardeola ralloides* ^*§*^	1	0.2	Ciconiformes	n	%	Suliformes	n	%
*Egretta alba*	1	0.2	*Ciconia ciconia*	121	100.0	*Phalacrocorax aristotelis*^*§*^	37	37.0
*Plegadis falcinellus*	1	0.2				*Morus bassanus*	34	34.0
Total	474	100.0	Piciformes	n	%	*Phalacrocorax carbo*	29	29.0
Apodiformes	n	%	*Picus viridis*	194	85.1	Total	100	100.0
*Apus apus*	7030	85.0	*Dendrocopos major*	22	9.6	Columbiformes	n	%
*Apus melba*	1214	14,7	*Jynx torquilla*	7	3.1	*Streptopelia decaocto*	349	58.7
*Apus pallidus*	28	0.3	*Dryocopus martius*	3	1.3	*Columba palumbus*	222	37.3
Total	8272	100.0	*Dendrocopos minor*	2	0.9	*Streptopelia turtur*	24	4.0
Galliformes	n	%	Total	228	100.0	Total	595	100.0
*Coturnix coturnix*	50	58.1	Coracciiformes	n	%	Cuculiformes	n	%
*Alectoris rufa*	35	40.7	*Merops apiaster*	117	57.9	*Cuculus canorus*	31	50.8
*Tetrao urogallus* ^*§*^	1	1.2	*Alcedo atthis*	77	38.1	*Clamator glandarius*	30	49.2
Total	86	100.0	*Coracias garrulus*	8	4.0	Total	61	100.0
Caprimulgiformes	n	%	Total	202	100.0			
*Caprimulgus europaeus*	298	68.0	Bucerotiformes	n	%	Otidiformes	n	%
*Caprimulgus ruficollis*	140	32.0	*Upupa epops*	139	100.0	*Tetrax tetrax* ^*§*^	1	100.0
Total	438	100.0						
**Birds**								
Passeriformes	n	%	Passeriformes	n	%	Passeriformes	n	%
*Carduelis carduelis*	10623	41.0	*Luscinia megarhynchos*	26	0.1	*Sylvia borin*	5	0.0
*Carduelis chloris*	3436	13.3	*Coccothraustes coccothraustes*	25	0.1	*Turdus iliacus*	5	0.0
*Carduelis cannabina*	2755	10.6	*Prunella modularis*	22	0.1	*Alauda arvensis*	4	0.0
*Fringilla coelebs*	1431	5.5	*Loxia curvirostra*	21	0.1	*Anthus triviallis*	4	0.0
*Passer domesticus*	1270	4.9	*Corvus corax*	17	0.1	*Lanius collurio*	4	0.0
*Delichon urbicum*	1001	3.9	*Phylloscopus trochilus*	15	0.1	*Lanius senator*	4	0.0
*Serinus serinus*	835	3.2	*Corvus corone*	14	0.1	*Muscicapa striata*	4	0.0
*Turdus merula*	778	3.0	*Parus cristatus*	14	0.1	*Phoenicurus phoenicurus* ^*§*^	4	0.0
*Pica pica*	634	2.4	*Turdus viscivorus*	12	0.0	*Sitta europaea*	4	0.0
*Carduelis spinus*	616	2.4	*Aegithalos caudatus*	11	0.0	*Sturnus unicolor*	4	0.0
*Sturnus vulgaris*	345	1.3	*Emberiza cia*	11	0.0	*Troglodytes troglodytes*	4	0.0
*Hirundo rustica*	264	1.0	*Parus ater*	11	0.0	*Acrocephalus arundinaceus*	3	0.0
*Garrulus glandarius*	198	0.8	*Melanocorypha calandra*	10	0.0	*Certhia brachydactyla*	3	0.0
*Parus major*	152	0.6	*Regulus ignicapilla*	10	0.0	*Hippolais opaca*	3	0.0
*Corvus monedula*	140	0.5	*Sylvia cantillans*	10	0.0	*Sylvia undata*	3	0.0
*Motacilla alba*	128	0.5	*Lullula arborea*	9	0.0	*Bucanetes githagineus*	2	0.0
*Erithacus rubecula*	118	0.5	*Saxicola torquatus*	9	0.0	*Calandrella brachydactyla*	2	0.0
*Turdus philomelos*	115	0.4	*Hippolais polyglotta*	8	0.0	*Locustella luscinioides*	2	0.0
*Sylvia atricapilla*	114	0.4	*Pyrrhocorax pyrrhocorax*	8	0.0	*Motacilla cinerea*	2	0.0
*Passer montanus*	73	0.3	*Sylvia communis*	8	0.0	*Saxicola rubetra*	2	0.0
*Oriolus oriolus*	66	0.3	*Emberiza schoeniclus*	7	0.0	*Serinus citrinella*	2	0.0
*Parus caeruleus*	65	0.3	*Galerida cristata*	7	0.0	*Anthus richardi*	1	0.0
*Emberiza cirlus*	61	0.2	*Petronia petronia*	7	0.0	*Certhia familiaris*	1	0.0
*Sylvia melanocephala*	53	0.2	*Regulus regulus*	7	0.0	*Emberiza citrinella*	1	0.0
*Fringilla montifringilla*	40	0.2	*Anthus pratensis*	6	0.0	*Monticola saxatilis*	1	0.0
*Phylloscopus collybita*	38	0.1	*Cettia cetti*	6	0.0	*Monticola solitarius*	1	0.0
*Pyrrhula pyrrhula*	38	0.1	*Emberiza calandra*	6	0.0	*Montifringilla nivalis*	1	0.0
*Acrocephalus scirpaceus*	33	0.1	*Cisticola juncidis*	5	0.0	*Ficedula albicollis*	1	0.0
*Ficedula hypoleuca*	30	0.1	*Hirundo daurica*	5	0.0	*Prunella collaris*	1	0.0
*Phoenicurus ochruros*	27	0.1	*Ptyonoprogne rupestris*	5	0.0	*Turdus torquatus*	1	0.0
						Total	25888	100.0
**Mammals**								
Insectivora	n	%	Rodentia	n	%	Lagomorpha	n	%
*Erinaceus europaeus*	1309	93.5	*Sciurus vulgaris*	353	89.6	*Oryctolagus cuniculus*	92	94.8
*Aetechinus (= Atelerix) algirus*	81	5.8	*Apodemus sylvaticus*	17	4.3	*Lepus granatensis*	5	5.2
*Crocidura russula*	8	0.6	*Eliomys quercinus*	11	2.8	Total	97	100.0
*Sorex araneus*	1	0.1	*Rattus norvergicus*	5	1.3			
*Suncus etruscus*	1	0.1	*Rattus rattus*	4	1.0	Chiroptera	n	%
Total	1400	100.0	*Glis glis*	3	0.8	*Pipistrellus pipistrellus*	506	57.8
			*Marmota marmota*	1	0.3	*Pipistrellus pipist*. *pygmaeus*	235	26.9
Carnivora	n	%	Total	394	100.0	*Pipistrellus kuhlii*	88	10.1
*Vulpes vulpes*	154	43.0	Artiodactyla	n	%	*Tadarida teniotis*	12	1.4
*Meles meles*	74	20.7	*Sus scrofa*	103	60.9	*Eptesicus serotinus*	10	1.1
*Martes foina*	66	18.4	*Capreolus capreolus*	48	28.4	*Microquiropterus sp*.	9	1.0
*Genetta genetta*	34	9.5	*Cervus elaphus*	9	5.3	*Plecotus auritus*	7	0.8
*Mustela nivalis*	21	5.9	*Rupricapra rupricapra*	7	4.1	*Miniopterus schreibersi*^*§*^	3	0.3
*Felis silvestris*	8	2.2	*Dama dama*	1	0.6	*Nyctalus leisleri*	3	0.3
*Mustela lutreola*^*§*^	1	0.3	*Ovis ammon*	1	0.6	*pipistrellus nathusii*	2	0.2
Total	358	100.0	Total	169	100.0	Total	875	100.0
**Amphibians**								
	n	%		n	%	Caudata	n	%
*Bufo calamita*	56	71.8	*Hyla arborea*	1	1.3	*Salamandra salamandra*	38	60.3
*Hyla meridionalis*	9	11.5	*Bufo viridis*	1	1.3	*Lissotriton helveticus*	10	15.9
*Bufo bufo*	7	9.0	*Rana iberica*	1	1.3	*Triturus marmoratus*	8	12.7
*Alytes obstetricans*	1	1.3	*Rana temporaria*	1	1.3	*Pleurodeles waltl*	7	11.1
*Discoglossus pictus*	1	1.3	Total	78	100.0	Total	63	100.0
**Reptiles**								
Squamata	n	%	Squamata	n	%	Squamata	n	%
*Malpolon monspessulanus*	133	33.4	*Natrix natrix*	7	1.8	Total	398	100.0
*Rhinechis scalaris*	114	28.6	*Vipera aspis*	5	1.3			
*Timon lepidus*	57	14.3	*Vipera latasti*	5	1.3	Testudines	n	%
*Chamaeleo chamaeleon*	23	5.8	*Zamenis longissimus*	4	1.0	*Testudo hermanni*^*§*^	1111	48.2
*Natrix maura*	21	5.3	*Coronella girondica*	2	0.5	*Mauremys leprosa*	747	32.4
*Anguis fragilis*	8	2.0	*Hierophis viridiflavus*	2	0.5	*Testudo graeca*^*§*^	414	17.9
*Tarentola mauritanica*	8	2.0	*Hemidactylus turcicus*	1	0.3	*Emys orbicularis*	35	1.5
*Hemorrhois hippocrepis*	7	1.8	*Lacerta viridis*	1	0.3	Total	2307	100.0

§ Menaced species according to the Spanish laws.

Birds accounted for 48633 (89%) admissions, followed by 3293 (6%) Mammals, 2705 (5%) Reptiles and 141 (0.3%) Amphibians. As regards to the sex, 16926 (31%) animals were males, 7865 (14%) females, and 29981 (55%) were undetermined. Within the male group, 10661 animals were finches, representing 63% of the males included in the study. As regards to age, 29549 (54%) of admissions were first calendar year animals, 16376 (30%) were >1 calendar year animals and 8874 (16%) were of undetermined age.

### Primary causes of morbidity /morbidity analysis

**Fig 1:**
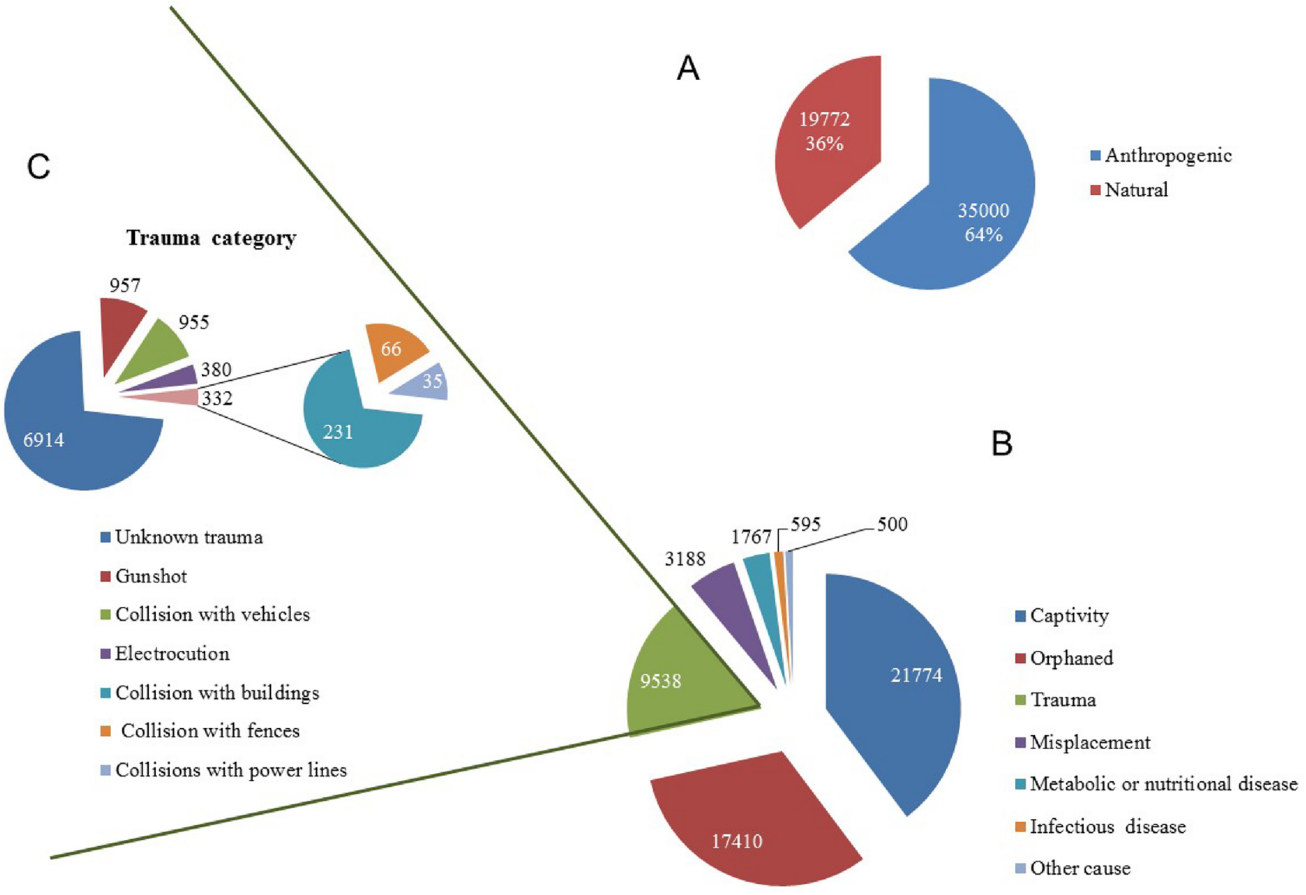
Primary causes of admission. A) Proportion of anthropogenic and natural causes. B) Absolute cases of primary causes of admission. C) Absolute cases of trauma category.

Anthropogenic interferences were involved in 64% of the admissions (Fig 1). “Captivity” was the most frequent cause of admission with 21774 animals [39.8%, (CI95%: 39.3–40.2)] in the overall period of study. Within this category, 75% of passerines and 73% of tortoises were the most frequently confiscated species (Table 2). “Orphaned” was the second most prevalent category with 17410 cases [31.8% (31.4–32.3)], mainly comprised by swifts (74%), rodents and rabbits (63%), and owls (57%). “Trauma” casualties were the third most important category with 9538 cases [17.4% (17.1–17.7)]; within this category, waders (71%), birds of prey (60%), herons and allies (59%), and carnivores (41%) presented the majority of the casualties (Table 2).

**Table 2:** Primary causes of admission expressed as a proportion within the animal group (rows). The “Undetermined” category was included in the “Other causes” due to the low number of cases (n = 58).

Animal Group	Total Admissions	Captivity		Orphaned		Trauma		Misplacement		Metabolic or nutritional		Infectious disease		Other cause	
	N	n	%	n	%	n	%	n	%	n	%	n	%	n	%
Amphibians	141	31	22.0	0	0.0	7	5.0	50	35.5	0	0.0	0	0.0	53	37.6
Chelonians	2307	1678	72.7	58	2.5	171	7.4	381	16.5	7	0.3	2	0.1	10	0.4
Squamata	398	22	5.5	8	2.0	87	21.9	271	68.1	5	1.3	2	0.5	3	0.8
Other aquatic birds	769	11	1.4	389	50.6	126	16.4	112	14.6	72	9.4	47	6.1	12	1.6
Herons	633	2	0.3	53	8.4	372	58.8	33	5.2	131	20.7	28	4.4	14	2.2
Waders	316	5	1.6	30	9.5	224	70.9	11	3.5	28	8.9	15	4.7	3	0.9
Marine birds	886	10	1.1	210	23.7	307	34.7	43	4.9	182	20.5	38	4.3	96	10.8
Owls	5209	136	2.6	2968	57.0	1486	28.5	341	6.5	192	3.7	38	0.7	48	0.9
Birds of prey	4910	266	5.4	823	16.8	2970	60.5	283	5.8	346	7.0	148	3.0	74	1.5
Swifts	8272	15	0.2	6153	74.4	1192	14.4	668	8.1	228	2.8	1	0.0	15	0.2
Other birds	1749	55	3.1	601	34.4	807	46.1	112	6.4	85	4.9	75	4.3	14	0.8
Passerines	25889	19470	75.2	4779	18.5	1107	4.3	257	1.0	170	0.7	62	0.2	44	0.2
Insectivora	1400	38	2.7	431	30.8	220	15.7	436	31.1	178	12.7	59	4.2	38	2.7
Carnivora	358	15	4.2	82	22.9	146	40.8	38	10.6	26	7.3	27	7.5	24	6.7
Rodents and rabbits	491	17	3.5	310	63.1	96	19.6	13	2.6	3	0.6	49	10.0	3	0.6
Artiodactyla	169	3	1.8	51	30.2	61	36.1	5	3.0	3	1.8	4	2.4	42	24.9
Chiroptera	875	0	0.0	464	53.0	159	18.2	134	15.3	111	12.7	0	0.0	7	0.8
Overall	54772	21774	39.8	17410	31.8	9538	17.4	3188	5.8	1767	3.2	595	1.1	500	0.9

Further analysis of the trauma category showed that 73% of the trauma were classified as of unknown origin (lack of information about the circumstances of the trauma or accident). Twenty-percent of traumas were due to gunshot (10%) and collision with vehicles (10%) (Fig 1). Diurnal birds of prey, herons and allies were the most affected by gunshot, representing 26.6% and 18.5% of cases respectively. Interestingly, 12% of the gunshot injured birds were admitted out of the hunting season. Within the category of collision with vehicles, mammals accounted for the higher proportion, especially affecting artiodactyls (52.5%) and carnivores (50.7%), followed by owls (22.5%).

Misplacement is an important reason of bringing to the Wildlife Rehabilitation Centers in reptiles (especially in squamata with a 68%) and hedgehogs (31%). Finally, note that in the miscellanea "Other" has been included causes with a very small number of cases, but relevant from the point of view of the impact of human activity, such as poisoning (25 cases), bycatch (37 cases) and oiled birds (54 cases).

Primary infectious and parasitic diseases included a wide variety of conditions. Thus, aspergillosis, trichomoniasis, coccidiosis and other endoparasites, avian poxvirus, and E. coli and Salmonella spp infections were the most common diseases diagnosed in birds. In mammals, the most prevalent diseases were myxomatosis in rabbits, sarcoptic mange in carnivores and parasitic pneumonia and abscesses in hedgehogs.

As regards to the distribution of cases along the calendar year, 48% were admitted in summer, 26% in spring, 15% in autumn and 11% in winter. The increase of cases during the spring and summer seasons was consequence of the onset of reports of orphaned animals which represented 38% and 48% of the admissions respectively. In autumn and winter, illegal captures (54–56%) and traumas (30–27%) were the most common causes of admission.

Year regression analyses showed a significant rise of the total number of attended animals along the years of study (R^2^ = 0.82; b = 271.55; p<0.001). Among them, orphaned (R^2^ = 0.89; b = 112.0; p<0.001), and animals suffering from trauma (R^2^ = 0.76; b = 22.3; p = 0.00), metabolic/nutritional (R^2^ = 0.71; b = 5.95; p<0.001) or infectious/parasitic diseases (R^2^ = 0.77; b = 3.95; p<0.001) had a significant increase. By contrast, in the trauma category, a slight decrease was observed in the collision with vehicles (R^2^ = 0.41; b = -1.63; p = 0.001); on the other hand, the slopes of regression for collision with buildings (R^2^ = 0.57; b = 1.07; p<0.001) and electrocution (R^2^ = 0.58; b = 0.95; p = 0.001) were very close to 1. No trend was observed in gunshot reports (R^2^ = 0.16; b = -0.09; p = 0.1).

### Outcome analyses

Overall, 7277 animals were euthanized (E_r_ = 13%), 12280 animals died during the rehabilitation process (M_r =_ 22%), 493 were kept in captivity (C_r =_ 1%), and 34722 animals were released (R_r_ = 63%).

**Fig 2:**
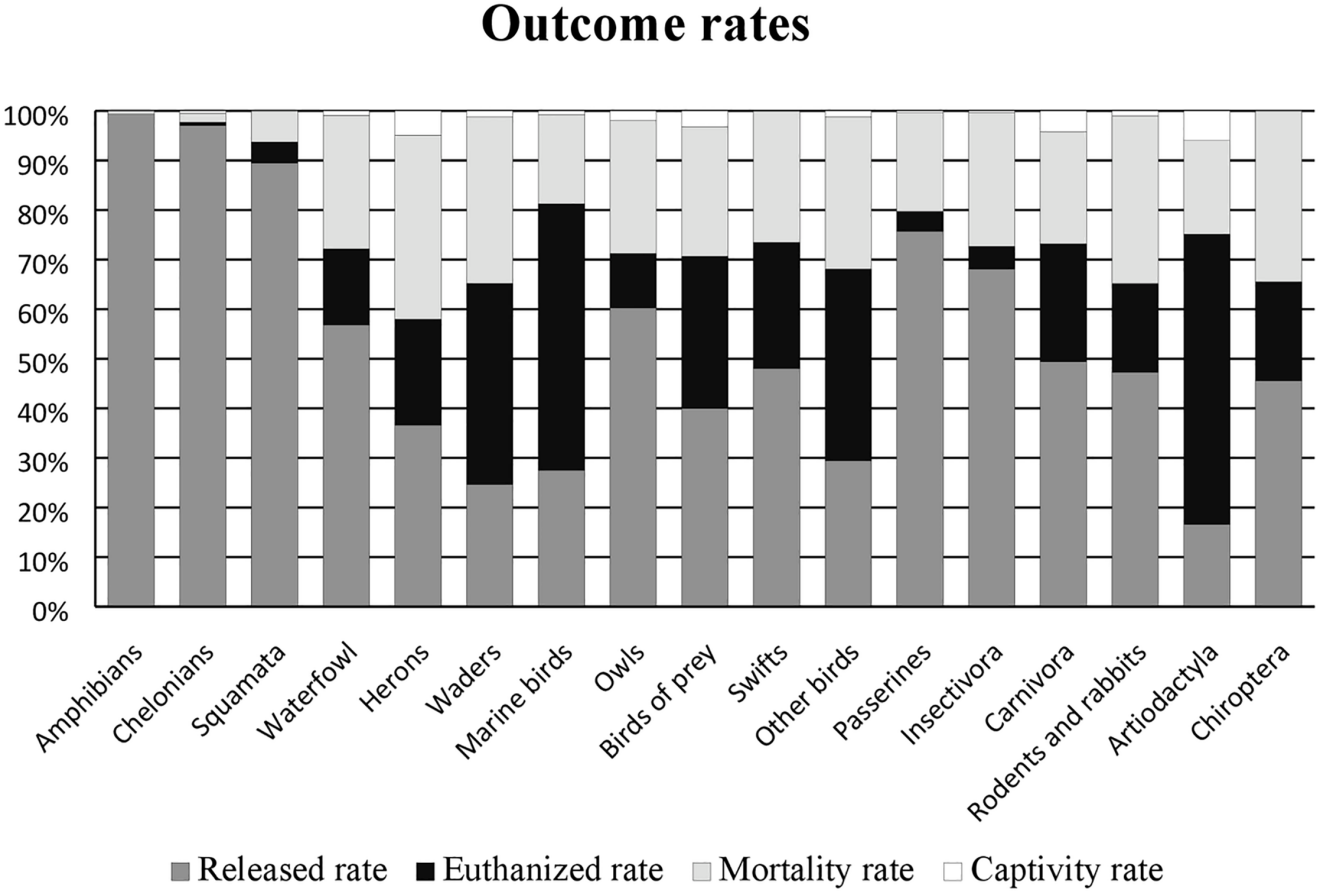
Outcome rates in the different zoological groups.

**Fig 3:**
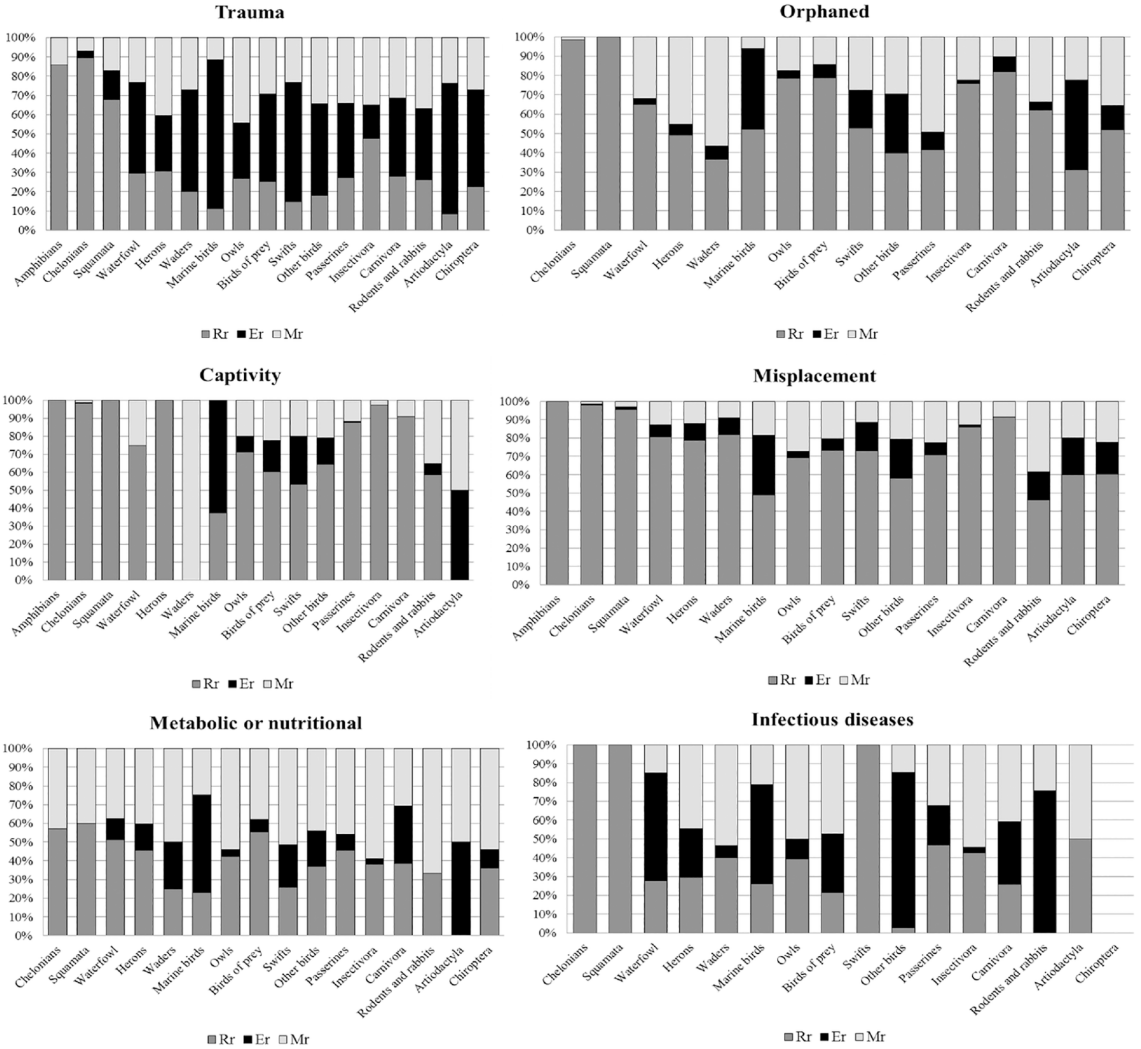
Outcome rates according to the zoological group in the different causes of admission. Rr = Released rate; Er = Euthanized rate; Mr = Mortality rate.

Outcome rates were different depending on the animal group and cause of admission. Marine birds (27.5%), waders (24.7%) and artiodactyla (16.6%) had the lowest release rate_,_ while amphibians and reptiles, as well as passerines presented the highest R_r_ above 75% (Fig 2). By contrast, the highest natural mortality (M_r_) was reported in the orphaned waders (56.7%), herons (45.3%), passerines (29.3%) and swifts (27.8%) (Fig 3).

When the outcomes rates were stratified by cause of admission and animal group, the “Captivity”, “Misplacement” and “Orphaned” categories showed the highest R_r_ (Fig 3). In the “Captivity” category scores above 85% were found in amphibians, reptiles, hedgehogs and passerines. In the “Orphaned” Rr above 75% were achieved in owls, diurnal birds of prey, hedgehogs and carnivores. On the other hand, the highest rates of mortality due to natural (Mr) or assisted (Er) death, were seen for “Trauma”, “Metabolic or nutritional” and “Infectious disease” (Fig 3).

**Fig 4:**
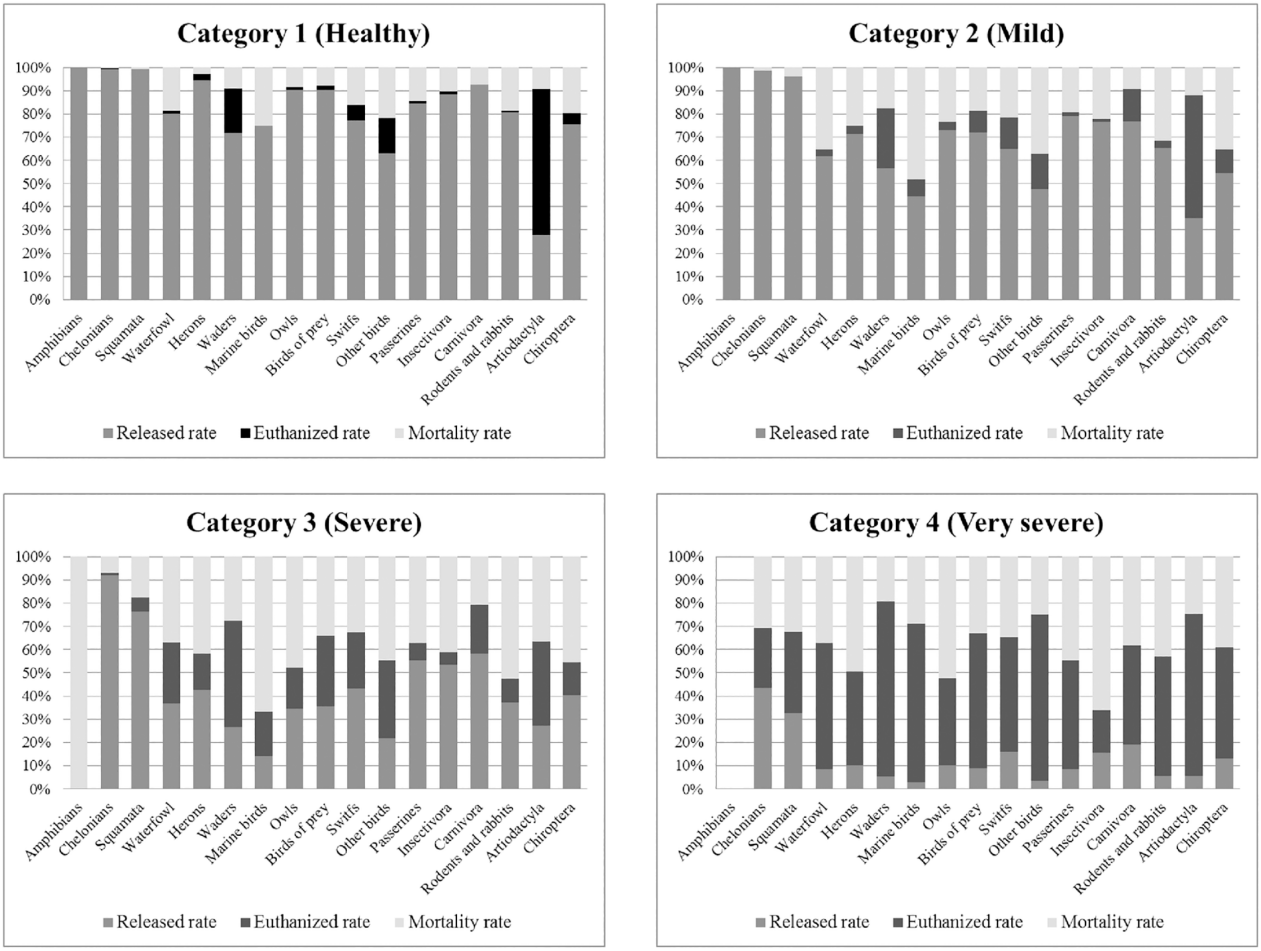
Outcome rates according to the zoological group in the different prognostic categories. Category description: 1, apparently healthy; 2, mild weakness or thinning, uncomplicated fractures; 3, severe (including dehydration, open fractures, deep wounds) and 4, very severe (major injuries, emaciation, paralysis, blindness, respiratory distress).

Outcomes have been also estimated stratified by taxonomic group and prognostic category (Fig 4). A higher release rate in the majority of taxonomic groups was observed in cases categorized as good prognosis (categories 1 and 2), with Rr values higher than 60%. In particular, reptiles have the highest release rates in all clinical categories. On the other hand, the Mr increases as the prognosis worsens. It should be noted that the groups with the highest Mr are seabirds and Chiroptera, especially in the categories with the best apparent prognosis (Fig 4). Finally, the Er was higher in categories 2 and 3, with values larger than 25%. In addition, the highest Er values were obtained in Artiodactyla, with observed values of 54%, 50%, 34.8% and 68.6% in the clinical categories 1 to 4, respectively (Fig 4).

### Cost-benefit estimator

Overall, the median T_s_ at the center was 9 days (P_10_ = 0; P_90_ = 69). The median days of stay at the centre was 17 days (P_10_ = 0; P_90_ = 80) for released animals, 3 days for natural death (P_10_ = 0; P_90_ = 40) and 0 days for euthanized (P_10_ = 0; P_90_ = 32).

In the group of released animals, the longest T_s_ were observed in diurnal birds of prey and owls with around two months of hospitalization (Table 3). In mammals, rodents and rabbits, hedgehogs and bats have T_s_ values around one and a half months. Similarly, when we consider the cause of admission, the overall process of rehabilitation of trauma casualties and the orphaned young took the longest median T_s_ (more than one month) and by contrast, misplacement had the shorter time (3 days). Trauma has the higher T_s_ (P_90_ = 284), independent of the zoological group. Importantly, hand-rearing orphans had median T_s_ between 20–60 days, with values around 20 days in swifts and other bird categories, which represent the largest number of chicks. On the other hand, both infectious diseases and metabolic and nutritional were associated with high recovery times, especially in hedgehogs, carnivore, owls and birds of prey. Finally, it was noteworthy the high T_s_ of birds of prey that have been kept illegally in captivity.

**Table 3:** Stay (days) at the center for released animals in the WRC of Torreferrusa.

Stay at the center	Trauma			Orphaned			Captivity			Misplacement			Metabolic/nutritional			Infectious			Others		
	P_10_	P_50_	P_90_	P_10_	P_50_	P_90_	P_10_	P_50_	P_90_	P_10_	P_50_	P_90_	P_10_	P_50_	P_90_	P_10_	P_50_	P_90_	P_10_	P_50_	P_90_
Amphibians	0	1	.				0	1	20	0	0	3	.	.	.	.	.	.	.	.	.
Chelonians	0	4	22	0	2	31	0	2	18	0	3	25	1	2	.	3	7	.	0	4	58
Squamata	0	1	5	0	0	.	0	1	6	0	0	4	0	2	.	0	1	.	1	3	.
Waterfowl	0	5	66	0	50	118	0	0	.	0	2	66	0	3	16	5	14	82	0	0	.
Herons	7	47	141	10	41	119	.	.	.	1	11	66	4	19	72	0	17	.	7	19	.
Waders	0	7	114	26	41	170	.	.	.	0	1	.	7	9	.	2	5	.	.	.	
Marine birds	0	28	78	3	49	88	1	2	.	0	5	67	0	13	50	4	14	47	1	19	84
Owls	16	104	442	21	66	175	16	128	474	0	28	212	11	57	249	31	131	623	2	65	349
Birds of prey	24	121	434	1	43	147	13	127	397	1	27	228	6	42	249	26	78	659	0	33	311
Swifts	0	1	32	2	19	42	0	27	.	0	0	25	0	6	21	.	.	.	0	3	.
Other birds	0	5	47	0	21	55	4	20	20	0	1	24	0	10	43	6	14	.	0	9	.
Passerines	0	11	52	5	26	68	0	12	57	0	2	42	0	8	58	0	16	48	1	11	67
Insectivora	16	44	124	12	49	166	1	19	181	3	28	132	16	50	160	21	48	93	14	58	237
Carnivora	5	33	121	0	22	183	4	38	171	0	13	60	12	27	90	16	32	.	0	3	143
Rodents and rabbits	0	51	156	11	54	152	0	11	78	0	13	.	.	.	.	.	.	.	.	.	.
Artiodactyla	0	27	.	0	19	113	.	.	.	0	1	.	.	.	.	.	.	.	1	2	.
Chiroptera	0	15	80	1	55	85	.	.	.	0	1	64	3	20	76	.	.	.	.	.	.

As regards of the cost-benefit index, the best values were observed in amphibian, reptiles and passerines, with values ranging from 4 to 5 released animals per euro and day (Table 4). On the other hand, when we consider the cause, the best results were obtained in the captivity (4.6 animals/euro/day) and misplacement (4.1) categories (Table 4). Interestingly the orphaned group represented 2.9 animals/euro/day, and the most efficient hand-rearing corresponded to raptors in birds, and Carnivora and Insectivora in mammals. On the other hand, the worst values were observed in the group of trauma casualties (1.3 animals/euro/day) and infectious diseases (1.4), especially in raptors, waders, marine birds and bats. In general, the cost-benefit index was higher in the cases with better prognosis (Table 5).

**Table 4:** Cost-benefit analyses of the rehabilitation process. The cost-benefit index express the number of released animals per euro and day of stay at the WRC.

*Animal group*	*Overall ratio*	*Trauma*	*Orphaned*	*Misplacement*	*Captivity*	*Infectious disease*	*Metabolic or nutritional*
*Amphibians*	5.2	4.5	na	5.3	5.3	na	na
*Chelonians*	5.1	4.7	5.2	5.1	5.2	5.3	3.0
*Squamata*	4.7	3.6	5.3	5.0	5.3	5.3	3.2
*Waterfowl*	3.0	1.5	3.4	4.1	2.9	1.5	2.7
*Herons*	1.9	1.5	2.6	4.1	2.6	1.5	2.4
*Waders*	1.3	1.1	1.9	4.3	na	2.1	1.3
*Marine birds*	1.4	0.6	2.7	2.6	1.6	1.4	1.2
*Owls*	3.2	1.3	4.1	3.6	3.4	2.1	2.2
*Birds of prey*	2.1	1.3	4.0	3.8	2.9	1.1	2.7
*Swifts*	2.5	0.8	2.8	3.8	2.8	5.3	1.4
*Other birds*	1.5	0.9	2.1	3.1	3.0	0.1	1.9
*Passerines*	4.0	1.4	2.2	3.7	4.6	2.5	2.4
*Insectivora*	3.6	2.5	4.0	4.5	5.1	2.2	2.0
*Carnivora*	2.6	1.4	4.0	4.4	3.5	1.4	2.0
*Rodents and rabbits*	2.5	1.4	3.2	2.4	3.1	0.0	1.8
*Artiodactyla*	0.9	0.4	1.4	3.2	na	2.6	0.0
*Chiroptera*	2.4	1.2	2.7	3.2	na	na	1.9
*Overall causes*	3.3	1.3	2.9	4.1	4.6	1.4	2.1

na, not applicable.

**Table 5:** Cost-benefit analyses of the rehabilitation process according to the prognostic category. The cost-benefit index expresses the number of released animals per euro and day stay at the WRC.

Prognosis category*	1(Healthy)	2(Mild)	3(Severe)	4(Very severe)
Amphibians	5.0	0.3	0.0	0.0
Chelonians	4.2	0.7	0.3	0.0
Squamata	3.9	0.7	0.4	0.2
Waterfowl	3.9	0.8	0.4	0.2
Herons	0.8	1.8	2.1	0.5
Waders	1.9	1.5	1.4	0.4
Marine birds	0.8	3.0	1.3	0.3
Owls	2.9	1.6	0.6	0.1
Birds of prey	1.5	1.9	1.5	0.4
Swifts	2.3	1.4	1.1	0.5
Other birds	2.4	1.7	0.9	0.2
Passerines	3.0	1.8	0.4	0.0
Insectivora	3.0	1.4	0.6	0.2
Carnivora	2.2	1.0	1.2	0.8
Rodents and rabbits	2.7	1.9	0.5	0.2
Artiodactyla	2.3	1.1	1.1	0.8
Chiroptera	2.1	1.9	0.9	0.4
Overall	2.9	1.6	0.6	0.2

*1, apparently healthy; 2, mild weakness or thinning, uncomplicated fractures; 3, severe (including dehydration, open fractures, deep wounds) and 4, very severe (major injuries, emaciation, paralysis, blindness, respiratory distress).

## Discussion

It is important to take into consideration that the purpose of the Wildlife Rehabilitation Centers (WRC) is the release of healthy animals to the appropriate habitats in the wild after a temporary care in captivity. For this reason, the evaluation of data about the rehabilitation practice is essential to have reference values for comparison purposes among different WRC in order to critically analyze the protocols and improve efficiency if necessary in each center. To our knowledge, the present epidemiological study is one of the largest and long-term studies conducted in a WRC. Data reported in this study should have a significant impact in the morbidity analysis as regards the large number of animals (>55000) and diversity of species (>300) included. Moreover, it provides new information about outcomes and the cost-benefit estimators of the rehabilitation process that can be useful as a reference for professionals involved in wildlife medicine and management.

It is well documented that anthropogenic factors are the most prevalent cause of admission in the WRC worldwide, representing up to 31% of the total admissions [11]. In this study, the most frequent cause of admission (40%) was the illegal confiscation of protected species, in particular of finches and tortoises. In Spain, trapping and confinement for leisure purposes (singing competition) of male birds of the family Fringillidae is a traditional activity. However, nowadays there are much more regulation that is restricted and more persecuted illegal captures. In Europe, the illegal taking and trading in wild birds is still a serious problem with clear regional patterns, having a considerable negative impact on biodiversity across the continent [23].

Illegal possession of reptiles, principally of tortoises for pet trade, is an important threat for species of the genus *Testudo* in the Mediterranean and Asia Minor regions [24,25]. In Spain, this trade has never reached the high levels observed in some other countries, although a regular national trade has been found within the natural range of the species around urban centers such as Madrid and Barcelona [26]. Moreover, in different areas of Spain, the capture of wild tortoise species to keep them as pets is a long-established tradition [27].

The second leading cause of admission in this study was the orphaned young, representing a 32% of the cases. This percentage of orphaned was very similar to the 28% reported in United Kingdom [28]; but higher than the 17% in Andalusia (Southern Spain) [29] and the 14% reported in Australia [9]. As previously described [10, 30], most of the attended cases belong to species living in close contact with urban and surrounding areas. Indeed, the Wildlife Rehabilitation Center is located in a densely populated region and the finding of juvenile wild animals is very common. Moreover, the social awareness on animal welfare and the information campaigns in the media have also contributed increasing the number of cases attended at the center along the period of study. Hand-rearing wildlife is a long, difficult, and expensive time-consuming task, although it can highly change depending of the species (owls are much more easy to rear than swift for instance). Many aspects should be considered critical for the success of the process, including both the physical development of healthy animals and the acquisition of natural behavior. On the other hand, the majority of those admissions are concentrated during the breeding season of these species along the summer and spring months, demanding an implementation of staff and economical resources management. Successful post-release survival rates of hand-reared wild animals have been reported in some species, justifying those efforts and expenses [31, 32, 33, 34].

Trauma related with anthropogenic activities represented another important cause of morbidity. In our study, the trauma of unknown origin represents the largest number of admissions and shows an increasing trend, compared to other causes of injury. Unfortunately, this result can be explained by errors in the identification and classification of the origin of trauma, which are intrinsic to the collection of information in the Wildlife Rehabilitation Centers. A more detailed analysis of the trauma category has confirmed that collisions with vehicles are the second leading cause of injury, especially in mammals and birds, as in other reports [11]. Gunshot was still present, indicating that, despite the legal protection of most of the species in Spain, illegal hunting has not been eradicated. In particular, shooting was relevant mainly in birds of prey, which have traditionally been considered competitors for humans [35].

Misplacement was especially important in amphibians, reptiles and Insectivora (mainly hedgehogs). In most of these cases, those animals are found in the proximity of human settlements or buildings. Similar to the young category, living near humans, increases the possibility of contact of these animals with the public, especially in densely populated areas [10].

Finally, the positive increase of the admissions due to primary infectious and metabolic diseases along the years of study might be consequence of the improvement in diagnostic and health protocols. In this kind of studies it is worthy to remark that mortality rates attributed to infectious or parasitic diseases or chronic poisoning may be underestimated, being a possible bias of the study. However, due to financial constraints at the WRC, we must assume such kind of bias since it is economically unaffordable a thorough analysis in all admitted cases.

In our study, the analysis of the rehabilitation outcomes showed an overall release rate (R_r_) over 50% of the admissions, higher than previously reported outcomes in other generalistic Wildlife Rehabilitation Centers. In a recent review, an overall R_r =_ 40% has been published in the centres of the RSPCA in UK [12]. Similarly, the Rr in Wildlife Rehabilitation Centers in Australia ranged from 38 to 45% [9]. The analysis of the rehabilitation outcomes showed that “Captivity”, “Misplacement” and “Orphaned” categories presented the best rate scores of releases. The highest R_r_ found in the “Captivity” and “Misplacement”, could be mostly explained by the large proportion of healthy animals, especially the recently captured birds. In fact, the severity of the clinical condition has been reported as the best predictor for the individual survival and release of wildlife casualties despite the species [36, 37]. The best Rr of the Orphaned young was seen for the raptors and owls and in the hedgehogs. These results are very similar to that described in other Wildlife Rehabilitation Centers [28, 38].

As regards as the M_r_ the overall value of 22% is lower than the 34% published in Australia [9]. As mentioned above, higher mortality is associated with the severity of injuries. For this reason, the highest Mr were observed in most groups of animals due to trauma, infectious and metabolic or nutritional diseases. On the other hand, the highest rates of natural death (Mr) were found in “Orphaned” waders and passerines. Hand-rearing of these birds results is a challenging task due to the heterogeneity of species and diets and the inherent fragility of the pediatric patients [39]. Many factors must be considered in order to address this problem such as the composition and preservation of food, hand-rearing and weaning protocols or prophylactic medical treatments; moreover, other additional difficulties in wildlife rehabilitation practices are obtaining necropsy specimens that are not autolytic and the budget constraints for postmortem studies. The overall value (11%) of Mr in the illegal captive category was lower than that reported in parrots in South America [40]. The mortality reported in that study of parrot trade was mainly consequence of massive confiscations of animals kept or transported improperly, and it comprised a mortality of 31% during transport, related to stress, sickness, rough handling and asphyxiation. Severe deficiencies in animal welfare are of major concern in wildlife trade [41].

Finally, it would be emphasized that euthanasia is the most reasonable decision when the animal’s welfare is compromised, due to the animal injuries or when the prognosis is poor or the animal unsuitable for release [1, 42]. In two retrospective studies performed in Australia, the E_r_ was 50% in Queensland [43], and 18% and 24% in Victoria and New South Wales, respectively [9]. In the present work, the overall E_r_ was lower; however, a stratified analysis of the data is necessary in order to compare the outcomes between centers. The higher proportion of euthanasia was observed in the trauma casualties, independent of the animal group. In fact, injuries that are associated with serious disabilities such as severe fractures, neurological deficits or soft tissue damage can lead to the decision to euthanize [11]. Although, we have not detailed the clinical signs of the patients included in this paper, it would be inferred that wing fractures or luxations in swifts and bats or soft tissue damage in marine birds are associated with a very poor prognosis. In the group of orphaned, the most critical aspects in the rehabilitation process is the acquisition of natural behavior and skills to survive in the wild. Moreover, assessing the degree of socialization is a difficult task. Unfortunately, wild animals suffering socialization problems or imprinting should not be released. The higher E_r_ has been observed in marine birds and artiodactyla. In fact, most of the Artiodactyla are considered as game species in Catalonia. In those cases, euthanasia considerations are based not only on the clinical prognosis and the individual welfare, but also taking into account biological hazards and economic criteria.

Importantly, the early assessment of prognosis and suitability for release is crucial in order to avoid unnecessary suffering of wildlife attended in Wildlife Rehabilitation Centers [44]. For this reason, the main goal of these Centers is to achieve the release as quickly and effectively as possible. In consequence, the time of stay could be a useful tool for the evaluation of Wildlife Rehabilitation Center. Unfortunately, this parameter is scarcely reported in the literature. In the mentioned work in Australia, 64% of the casualties have a time of stay (T_s_) between 0–5 days and 7% stay more than 100 days [9]. In our experience, the media T_s_ is 9 days and the P_90_ is 69 days for the overall cohort. Due to the non normal distribution of this variable, this variable should be presented as median and percentiles.

Moreover, in our study, the T_s_ was introduced as an estimator of the cost of the rehabilitation process, since each day of hospitalization in the center represents a cost in staff, food and medicines. Although this is not a complete measure of the real cost, this parameter can be an indication of resource usage, and be useful as a rough approach to efficiency [44]. Taken into account this concept, passerines represented the group with the lowest cost per animal released (T_s_ of 12 days). By contrast, poached birds of prey had the longest stay (128 days), mostly due to plumage and behavior abnormalities. Orphaned young passerines and swifts had also shorter stays than birds of prey and owls. Indeed, swifts represented 20% of the admitted hand-reared birds with a median stay of 19 days and a P_90_ of 40 days. Within the overall group of the orphaned young, the P_90_ was higher than 5 months as a result of the management policies of the center which does not allow releasing young animals during the winter months, especially mammals as bats and hedgehogs. Finally, trauma-related casualties were in general time-consuming, with long T_s_ and in consequence less efficient saving costs; in birds of prey were especially long (T_s_ = 114 days of median) because of the muscle-skeletal and nervous system injuries requires long clinical healing and rehabilitation. Similarly, conditions as infectious or metabolic diseases are also associated to long recovery times.

Economic evaluation (EA) is a quantitative technique developed by economists to promote the most efficient use of the resources. In human medicine, there are different studies of EA, as cost-effectivity, cost-utility or cost-benefit [45]. The cost and benefits associated with oiled bird care has been discussed, but those analyses are still scarce in WRC [46]. In the present study, we used a cost-benefit study in order to compare the effectiveness of the rehabilitation process according to the cause of admission and zoological group and prognostic category. The cost-benefit index revealed the worse results in the trauma casualties and infectious diseases, but also a low value in the orphaned group.

One of the most important limitations in this study was to assume that the daily cost was the same for the different species, clinical conditions and husbandry protocols. Although this approach is not accurate, allows an overall success estimation of the rehabilitation process and the detection of differences between zoological groups and admission categories. Nevertheless, our analyses must be considered partial because we did not perform comparisons among different alternatives of health or rehabilitation protocols, according to the specificities of the causes of admission and the diversity of species. A correction factor for the cost-benefit parameter should be introduced in further studies to compensate for cost differences depending on the species or taxonomic categories.

Finally, in Catalonia, the Wildlife Service is the only one that has competence in the care of wild species that are found injured or orphaned. For this reason most of the animals have been collected by the competent authorities from the wild or most of the time they picked up from citizens home. It should be noted that private citizens are the following group bringing animals directly to the WRC, as expected in an area so populated as the area of influence of the our center.

In our opinion, the cost-benefit analysis of wildlife rehabilitation based on the admission causes and the prognostic category are complementary and useful for the detection of critical points in the clinical and husbandry protocols and the management of WRC. In conclusion, we suggest that an initial approach to cost-effectiveness studies of Wildlife Rehabilitation Centers should include both the outcomes indicators, the stay at the center and a cost-benefit index in the different zoological groups and primary cause of admission. In the future, it would be desirable to conduct more specific cost-effectiveness analysis to improve the overall performance of rehabilitation, both for economic reasons and in order to improve the animal welfare.
